# Fabrication and characterization of microstructure-controllable COL-HA-PVA hydrogels for cartilage repair

**DOI:** 10.1007/s10856-021-06577-9

**Published:** 2021-08-18

**Authors:** Jie Xie, Wu Wang, Ruibo Zhao, Wei Lu, Liang chen, Weiping Su, Min Zeng, Yihe Hu

**Affiliations:** Department of Orthopaedics, Xiangya Hospital, Central South University, Changsha, Hunan China

## Abstract

Polyvinyl alcohol (PVA) hydrogel has gained interest in cartilage repair because of its highly swollen, porosity, and viscoelastic properties. However, PVA has some deficiencies, such as its poor biocompatibility and microstructure. This research aimed to design novel hydroxyapatite (HA)-collagen (COL)-PVA hydrogels. COL was added to improve cell biocompatibility, and the microstructure of the hydrogels was controlled by fused deposition modeling (FDM). The feasibility of the COL-HA-PVA hydrogels in cartilage repair was evaluated by in vitro and in vivo experiments. The scanning electron microscopy results showed that the hybrid hydrogels had interconnected macropore structures that contained a COL reticular scaffold. The diameter of the macropore was 1.08–1.85 mm, which corresponds to the diameter of the denatured PVA column. The chondrocytes were then seeded in hydrogels to assess the cell viability and formation of the cartilage matrix. The in vitro results revealed excellent cellular biocompatibility. Osteochondral defects (8 mm in diameter and 8 mm in depth) were created in the femoral trochlear of goats, and the defects were implanted with cell-seeded hydrogels, cell-free hydrogels, or a blank control. The in vivo results showed that the COL-HA-PVA hydrogels effectively repaired cartilage defects, especially the conditions inoculated with chondrocyte in advance. This research suggests that the COL-HA-PVA hydrogels have promising application in cartilage repair.

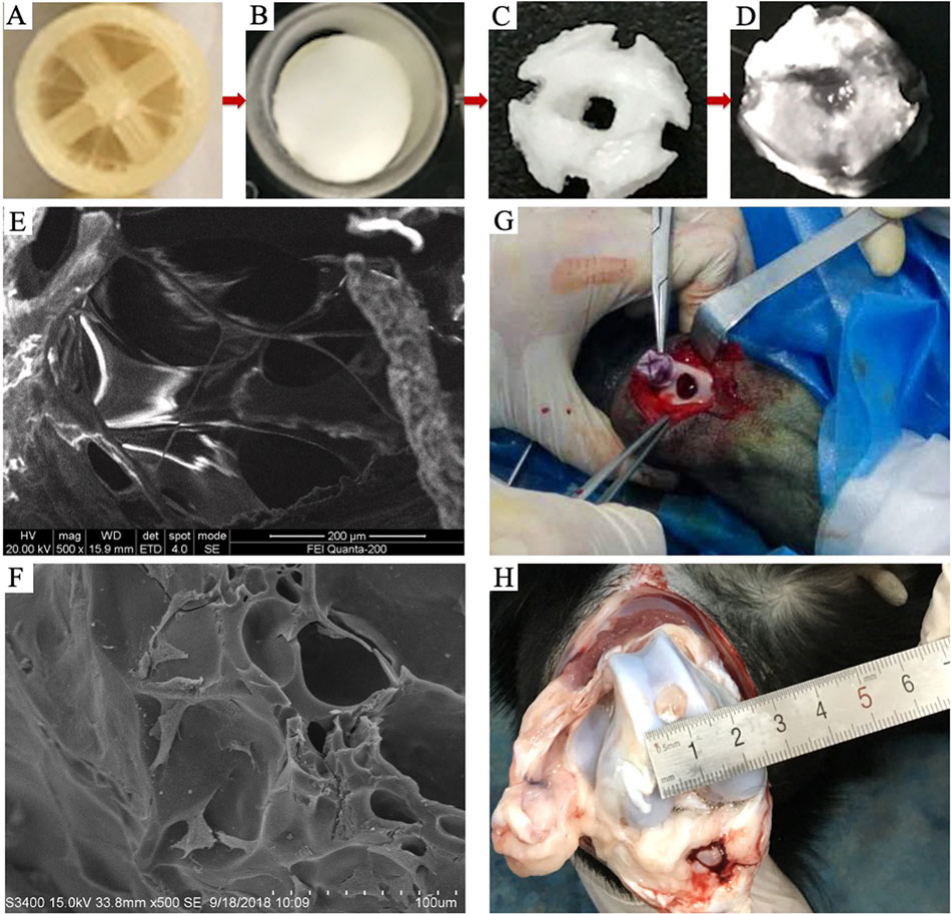

## Introduction

Cartilage injury has become a major public health problem because of the aging population and an increase in accident injuries [1–3]. Cartilage damage often results in an underlying bone defect and subsequently, joint dysfunction, because of the absence of vasculature and limited proliferative capacity of native cartilage [4, 5]. One strategy of growing interest in cartilage repair is artificial cartilage substitutes [6–8]. Numerous studies have demonstrated that hydrogels, such as a polyvinyl alcohol (PVA) hydrogel, have potential as repair materials because of their ability to swell, porosity, and viscoelastic properties [9–12]. However, the poor biocompatibility and limited mechanical property of a pure PVA hydrogel restrict its further application [13, 14]. Previous studies have indicated that hydroxyapatite (HA) and collagen (COL) can be added to a PVA hydrogel to improve the mechanical properties and biocompatibility of the hydrogel [15, 16]. Chen et al. constructed a polyacrylic acid (PAA)/PVA-HA composite hydrogel with the mechanical property that improved with the addition of HA particles [17]. Bates et al. added a COL-mimicking peptide to the PVA hydrogel and found that the COL-modified PVA encourages cell attachment while maintaining its compatibility [18]. In addition, our previous research indicated that the mechanics of a novel modified HA/PVA hydrogel were similar to those of native cartilage [19].

The uncontrollable macrostructure and poor microporous structure of a pure PVA hydrogel prepared using a routine method typically result in low integration with cartilage defects and have limited cartilage ingrowth [20, 21]. In addition, a cartilage substitute may be difficult to biologically integrate with peripheral native cartilages, thus leading to implant loosening. Three-dimensional printing (3DP), also known as rapid prototyping and additive manufacturing, can be used to regulate the three-dimensional structure of the hydrogel [7, 22, 23]. Liu et al. designed a porous chitosan (CS)/PVA hydrogel by 3DP for tissue regeneration [24]. Meng et al. revealed that an enhanced mechanical property of the PVA hybrid hydrogel is achieved by 3DP, which suggests a promising potential in the field of cartilage repair [7].

This study used fused deposition modeling (FDM) printing technology, a type of 3DP, to mold a new hybrid PVA hydrogel. Though hydrogels are typically denatured at high temperature because of thermal instability, which prevents gel formation, we found that the denatured PVA could be completely dissolved in double steamed water. In this study, we added a PVA solution to a denatured PVA mold, and the compound PVA was successively processed by melt freeze-dry crosslinking and double steam water demolding. New PVA hydrogels were formed, and the macro-microstructure of the porous PVA hydrogel could be controlled by computer-aided design (CAD). The general structure of the hydrogel can be precisely customized to make the artificial cartilage perfectly fit the defective surface, and the microstructure facilitates material exchange and cell ingrowth. In addition, COL and HA components were added to PVA scaffolds, simulating the natural extracellular matrix components and improving the mechanical properties, respectively. The macropores of the PVA hydrogel contain a COL reticular structure, which favors cell adhesion and subsequently, cartilage ingrowth. In the hybrid PVA hydrogels, the HA-PVA hydrogels simulate the characteristics of the viscoelastic material of natural cartilage and provide mechanical support, while the filled, degrading cell-loaded COL microscaffolds allow for neo-tissue formation and integration with peripheral native cartilage. The application possibility of the COL-HA-PVA hydrogels was evaluated through a series of in vitro and in vivo experiments.

## Materials and methods

### Fabrication and evaluation of COL-HA-PVA hydrogels

#### COL-HA-PVA hydrogels preparation

**Fig. 1:**
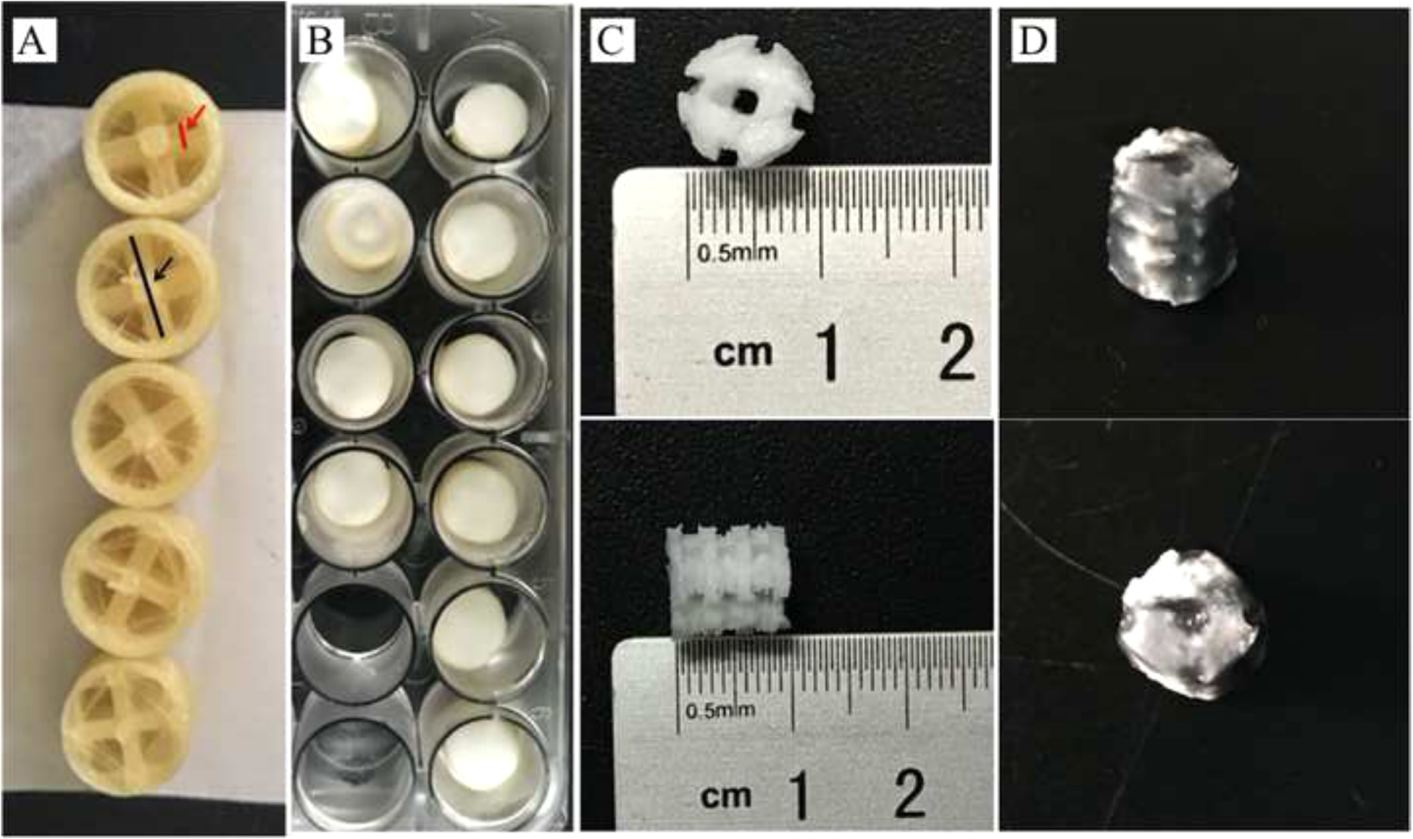
COL-HA-PVA hydrogels preparation: **A** denatured PVA mold, the thickness of the PVA column (red arrow) was 1.2, 1.4, 1.6, 1.8, and 2.0 mm, respectively, and the diameter of the denatured PVA mold (black arrow) was 8 mm; **B** compound PVA including denatured PVA mold and HA-PVA emulsion; **C** HA-PVA hydrogels; **D** COL-HA-PVA hydrogels

Using FDM technology, PVA powder was melted in the printing head, and the thermoplastic PVA was arranged with a specific protocol, which contributes to the formation of the denatured PVA mold. The cylinder PVA scaffold has a diameter of 8 mm and a height of 8 mm, and the diameter of the PVA thread in the printing head was 1.2, 1.4, 1.6, 1.8, or 2.0 mm, respectively, which provided the basis of grouping for further experiments (Fig. 1A).

The HA-PVA hydrogels were prepared through repeated freeze-drying and molding (Fig. 1B, C). Specifically: (1) mechanical blending of 15 wt% PVA and 5 wt% HA powder in double-distilled water with a magnetic stirrer [25, 26]; (2) transferring the HA-PVA emulsion into the denatured PVA mold to form a mixed structure; (3) freezing the mold/mixture at −20 °C for 10 h and thawing for 2 h at room temperature, repeating the process five times; (4) immersing the mixture in double-distilled water and stirring continuously to dissolve the denatured PVA mold; (5) freeze-drying the HA-PVA hydrogels for 24 h (−80 °C, 0 MPa).

The COL-HA-PVA hydrogels were prepared with freeze drying using genipin as a cross-linking agent (Fig. 1D). Specifically: (1) immersing the HA-PVA hydrogels in a 5 mg/ml COL I solution and centrifuging for 5 min to completely impregnate the pores of the hydrogels; (2) freezing the composite for 24 h at −20 °C and 12 h at −80 °C, and then freeze-drying for 24 h in a freeze dryer (−80 °C, 0 MPa), which forms the COL microscaffold in the HA-PVA hydrogels; (3) immersing the COL-HA-PVA composites into 0.5% genipin for 12 h to improve the cross-linking stability, and subsequently rinsing with 75% alcohol and double-distilled water to remove the residual genipin. Previous research revealed that gamma radiation is an effective method to sterilize PVA hydrogels and can further increase the crosslinking of the hybrid hydrogels [27]. The COL-HA-PVA hydrogels in our study were sterilized by gamma radiation using Cobalt-60 (25 KGy, 24 h).

#### Characterization of COL-HA-PVA hydrogels

Macro-microstructure characterizations of the hybrid PVA hydrogels were conducted in the gel state using environmental scanning electron microscopy (ESEM), with a beam spot, low vacuum mode (0.75 Torr), and high pressure (20 kV). The ESEM images were imported into Nano Measure software, and the diameters of the macropores were measured using a linear tool.

The moisture content of the hybrid hydrogels was measured by a weighing method. Specifically: the hybrid hydrogels were dried to a constant weight, recorded as W1; the dried sample was immersed in double-distilled water to reach a constant weight again, recorded as W2. The moisture content of the hybrid hydrogels was calculated by the following formula.

Moisture content = (W2–W1)/W2 × 100%

#### In vitro studies of COL-HA-PVA hydrogels

The articular cartilages of goats were collected in a sterile tube and stored in a PBS solution containing penicillin (10,000 U/ml) and gentamicin (5000 U/ml) for 6 h. The cartilage was cut into fragments of ~1 × 1 × 1 mm^3^ using a surgical blade and rinsed with PBS solution three times, digested with trypsin (25%, 5 ml) for 30 min and digested with type II collagenase (0.2%, 5 ml) for 6 h. The cell suspension was incubated in a humidified incubator (37 °C, 5% CO_2_) with chondrocyte basal medium (), and the medium was changed every 2 days until the chondrocytes were expanded to 90% confluency for further subculture. The density of the cell suspension was adjusted to 1 × 10^6^/mL in the in vitro studies.

The diameter of the PVA thread (1.2, 1.4, 1.6, 1.8, and 2.0 mm) provided the basis of the grouping for the experiments in vitro. The sterilized hydrogels were washed with PBS solution. The cell suspension (1 × 10^6^/mL, 100 μL) was then added to the sample and incubated at 5% CO_2_ and 37 °C for 1 h. The chondrocyte culture medium (500 ml of basal medium, 25 ml of FBS, 5 ml of Chondrocyte Growth Supplement, and 5 ml of penicillin/streptomycin solution) was then added to cover the hydrogels. The cell viability was studied by the cell adhesion rate on the first day, and the relative growth rate (RGR) and ESEM observations on the third day, according to the protocol of previous studies [9, 28]. HA-PVA hydrogels (without COL microscaffolds) prepared by the above method were used as the control group, and a chondrocyte plate culture was used as the Blank group for RGR analysis. On the seventh day in vitro, these samples were fixed in 4% paraformaldehyde for 24 h, and then embedded with paraffin for histological detection. The paraffin section was stained with hematoxylin and eosin (H&E), and COL II was detected using immunohistochemistry (IHC), according to the manufacturer’s protocol (Cyagen US, Inc). Histological analyses were performed using light microscopy.

#### In vivo studies of COL-HA-PVA hydrogels

Based on the results of the cell viability in vitro, ingrowth of chondrocytes was observed in all of the hydrogels. When the diameter of the denatured PVA column was 1.6 mm, the hydrogels were more capable of cartilage ingrowth. Therefore, the COL-HA-PVA hydrogels with a PVA column of 1.6 mm were used for further in vivo experiments.

**Fig. 2:**
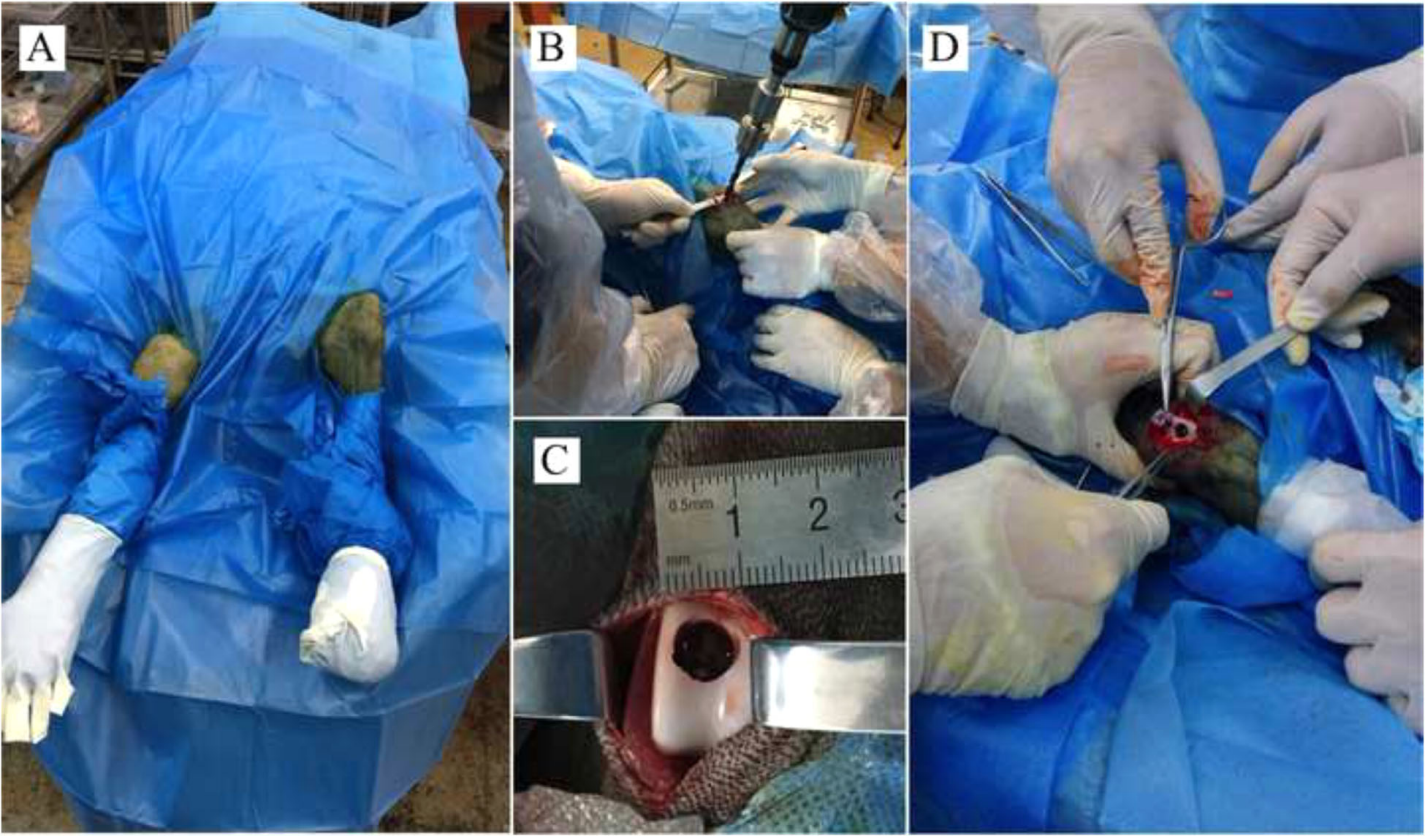
Animal model preparation: **A** shaving and sterilizing around incision; **B**, **C** Osteochondral defects were created by an electric drill; **D** hydrogels implantation

Ten 10-month-old goats were anesthetized intramuscularly (Sumianxin II, 0.1 ml/kg). Both knee joints were exposed via a medial parapatellar approach after shaving and sterilizing around the incision (Fig. 2A). Osteochondral defects (8 mm in diameter and 8 mm in depth) in the femoral trochlear were created by an electric drill (8 mm in diameter and 8 mm in depth) (Fig. 2B, C). The cell-seeded (allogeneic cells) and cell-free hydrogels were implanted into the defects separately (Fig. 2D). The defects of the control group did not receive implants, except for routine suture. The goats returned to normal activity after recovery from anesthesia and received intramuscular injection of antibiotics for 3 days after operation (penicillin, per day: 100,000 U/kg). Wound conditions were recorded regularly. One month after operation, the goats were sacrificed, and the resected distal femur was collected for examination. Half of the samples were embedded in paraffin, and stained with H&E, Safranin O, and Toluidine Blue. The remaining samples were used to measure the content of COL1I and GAG by spectrophotometry. The samples were exposed to dimethylmethylene blue, and the relative expressions of COL1A2, AGGRECAN, and SOX9 were detected by RT-qPCR.

#### Statistical analysis

SPSS19.0 software was used for statistical analysis. The data were recorded as mean ± standard deviation (X ± S). In accordance with normal distribution and homogeneity of variance, one-way ANOVA was used for data comparison among the groups, and an LSD test was used for pairwise comparison. Tamhane’s T2 test was used when the variance was uneven. Statistical difference was identified as *p* < 0.05.

## Results

### Characterization of the COL-HA-PVA hydrogels

**Fig. 3:**
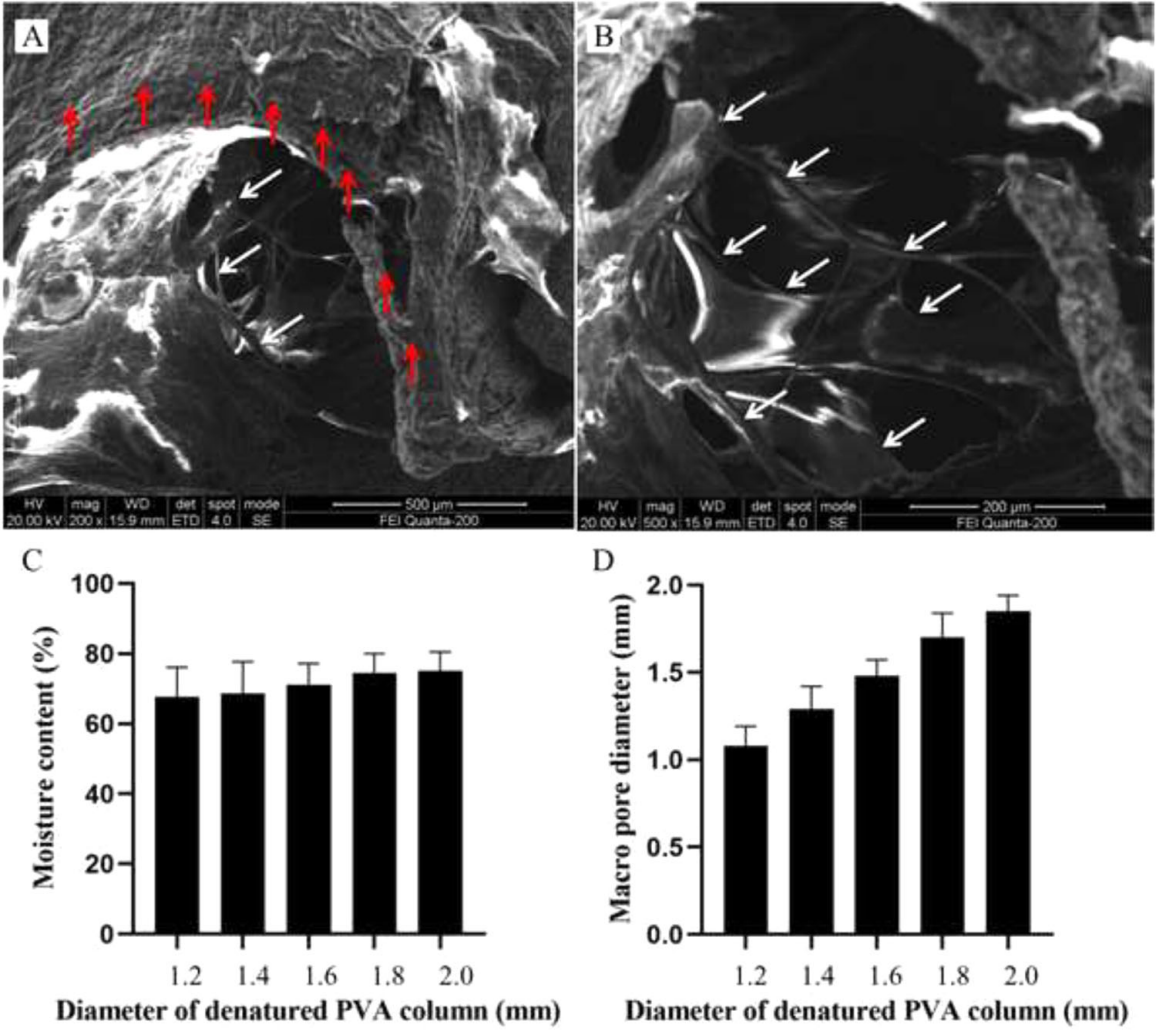
Characterization of COL-HA-PVA hydrogels: **A**, **B** microstructure of the COL-HA-PVA hydrogels by ESEM, the macropores of HA-PVA hydrogels (red arrow) contained COL reticular structure (white arrow); **C** moisture content of COL-HA-PVA hydrogels; **D** macropore diameter formed by HA-PVA hydrogels

The structures of the COL-HA-PVA hydrogels are demonstrated in Fig. 3A, B. The hybrid hydrogels have an interconnected macropore structure, and the macropores contained a COL reticular structure. As shown in Fig. 3C, the moisture content of each group was greater than 65% (range, 67.6–75.2%). With an increase in denatured PVA, the moisture content increased; however, there was no significant difference among the groups (*p* > 0.05). The diameter of the macropore was slightly smaller than that of the inverted PVA column (range, 1.08–1.85 mm) (Fig. 3D), and the size of the macropore generally corresponds to the diameter of the denatured PVA column.

### In vitro studies of COL-HA-PVA hydrogels

**Fig. 4:**
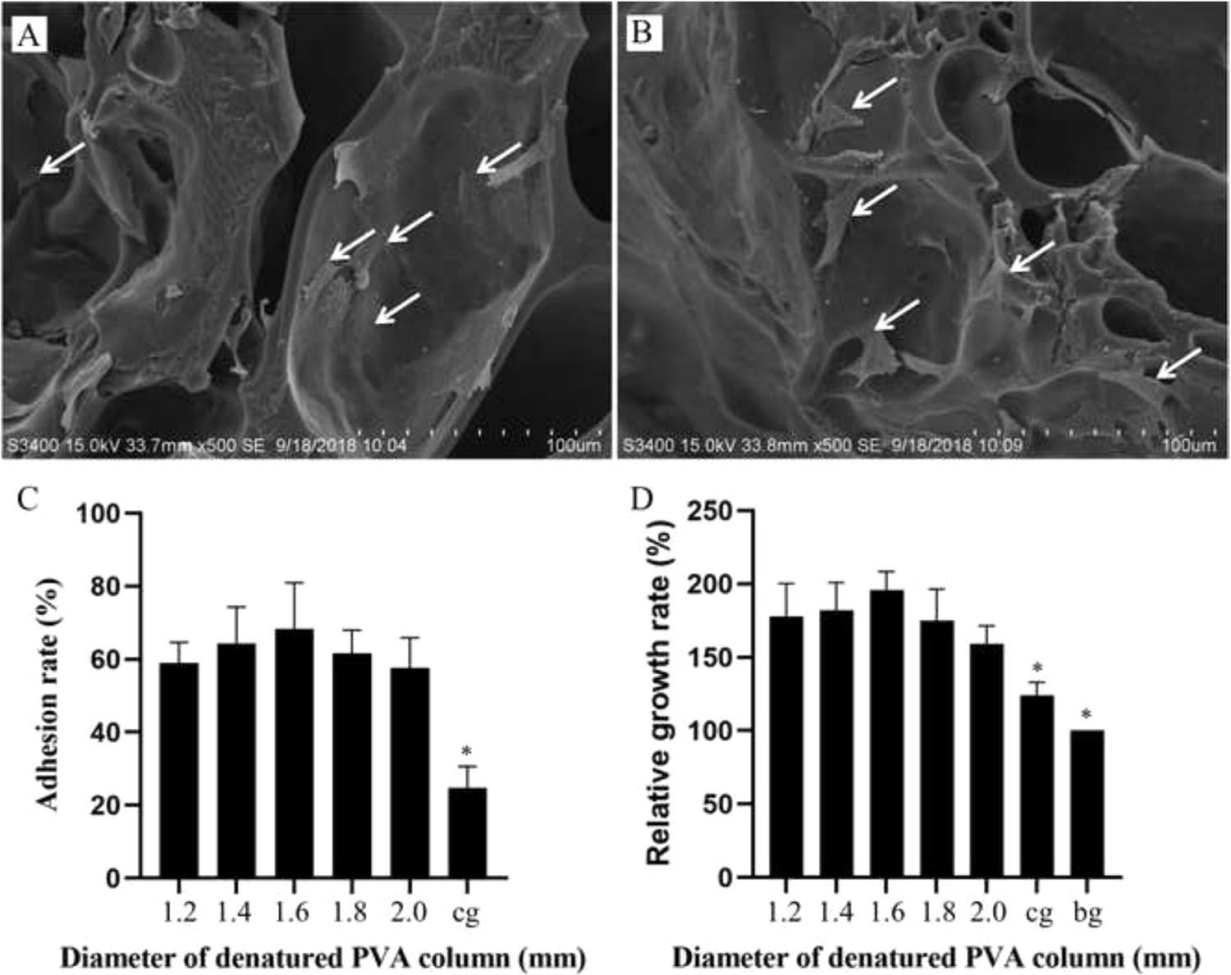
Cell viability of COL-HA-PVA hydrogels in vitro: **A**, **B** microstructure of the COL-HA-PVA hydrogels by ESEM on the 3rd day of culture, chondrocytes widely attach to COL reticular structure (white arrow); **C** adhesion rate of COL-HA-PVA hydrogels; (**D**) RGR of COL-HA-PVA hydrogels. (cg control group; bg: blank group; *p < 0.05, compared with hydrogels group)

**Fig. 5:**
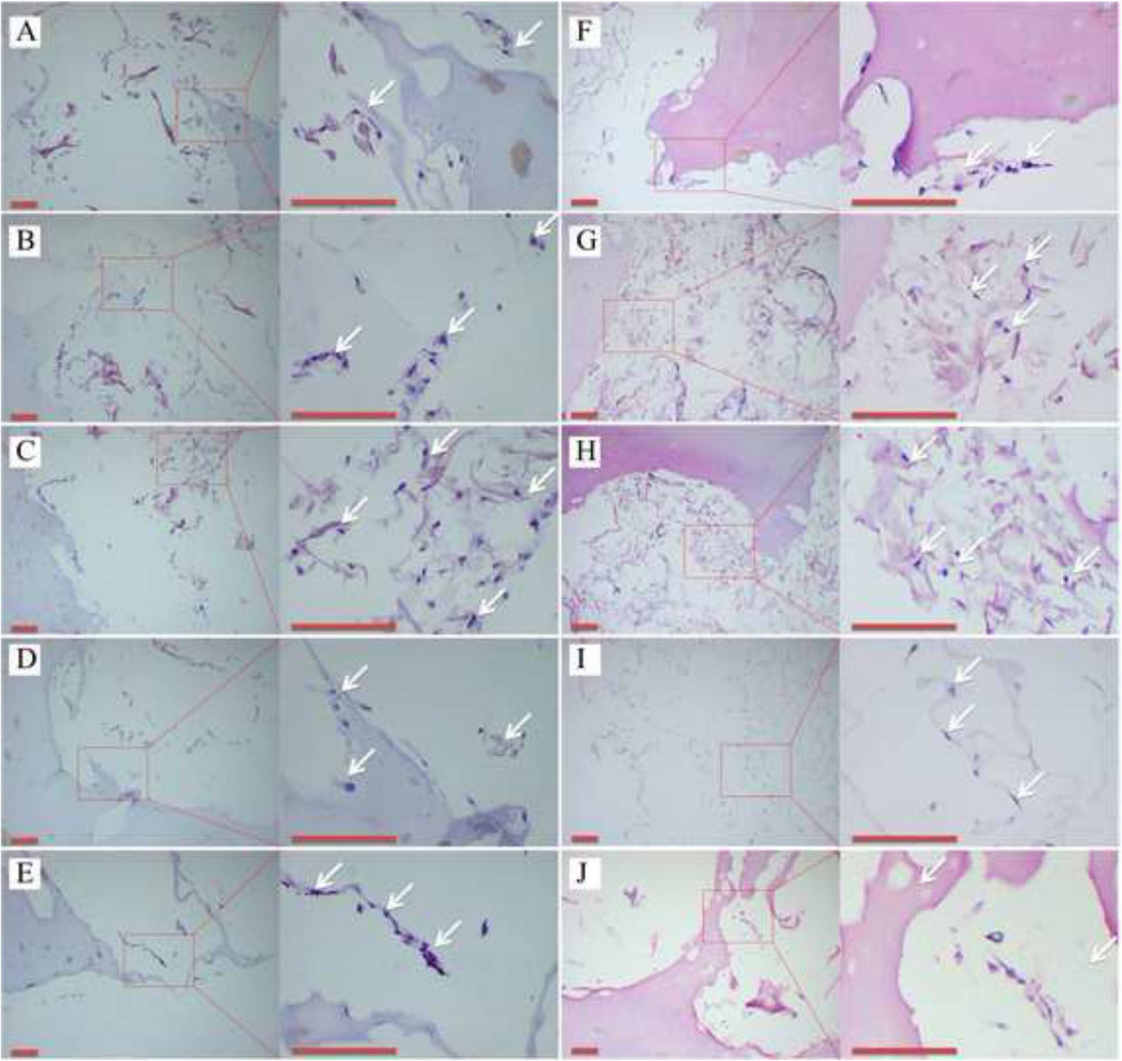
Representative H&E positive staining (white arrow) in vitro (scale bar: 100 mm): **A–E** the diameter of PVA thread was 1.2, 1.4 1.6, 1.8, and 2.0 mm, respectively, after 7 days of culture; **F–J** the diameter of PVA thread was 1.2, 1.4, 1.6, 1.8, and 2.0 mm, respectively, after 14 days of culture

**Fig. 6:**
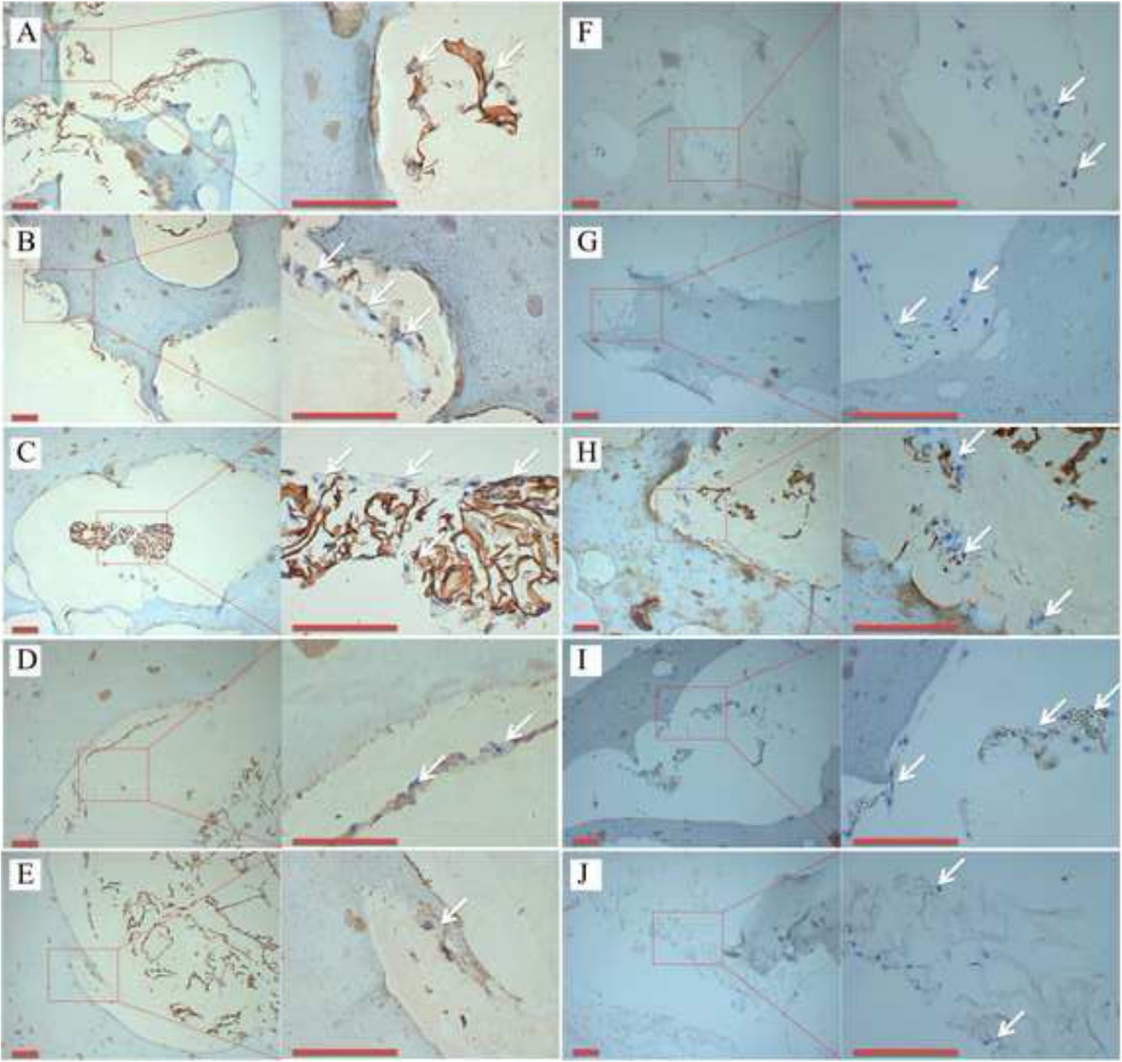
Representative IHC staining of COL II displayed brown yellow or brown particles (white arrow) in vitro (scale bar: 100 mm): **A–E** the diameter of PVA thread was 1.2, 1.4, 1.6, 1.8, and 2.0 mm, respectively, after 7 days of culture; **F**–**J** the diameter of PVA thread was 1.2, 1.4, 1.6, 1.8, and 2.0 mm, respectively, after 14 days of culture

As shown in the ESEM images (Fig. 4A, B), chondrocytes were uniformly attached to the COL reticular structure. The adhesion rate and RGR of the hybrid hydrogels containing the COL reticular structure were significantly greater than that in the control group without COL (*p* < 0.05), and there were no significant differences among the COL-HA-PVA hydrogels (*p* > 0.05) (Fig. 4C, D). After 7 and 14 days of culture, H&E staining exhibited new cartilage-like tissue formation in the macropores of HA-PVA hydrogels (Fig. 5), and IHC staining suggested the formation of cartilage-specific COL II (Fig. 6). Compared with the results of the 7-day culture, there was stronger staining of H&E and COL II at 14 days. The proliferation of chondrocytes and production of cartilage matrix were greatest when the diameter of the print-denatured PVA scaffold was 1.6 mm.

### In vivo studies of the COL-HA-PVA hydrogels

**Fig. 7:**
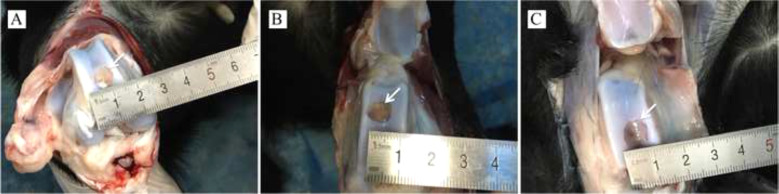
General observation of defects, new cartilage-like tissue (white arrow) around the defect was visible: **A** COL-HA-PVA hydrogels with the cell-seeded group; **B** COL-HA-PVA hydrogels with cell-free group; **C** the control group without hydrogels

**Fig. 8:**
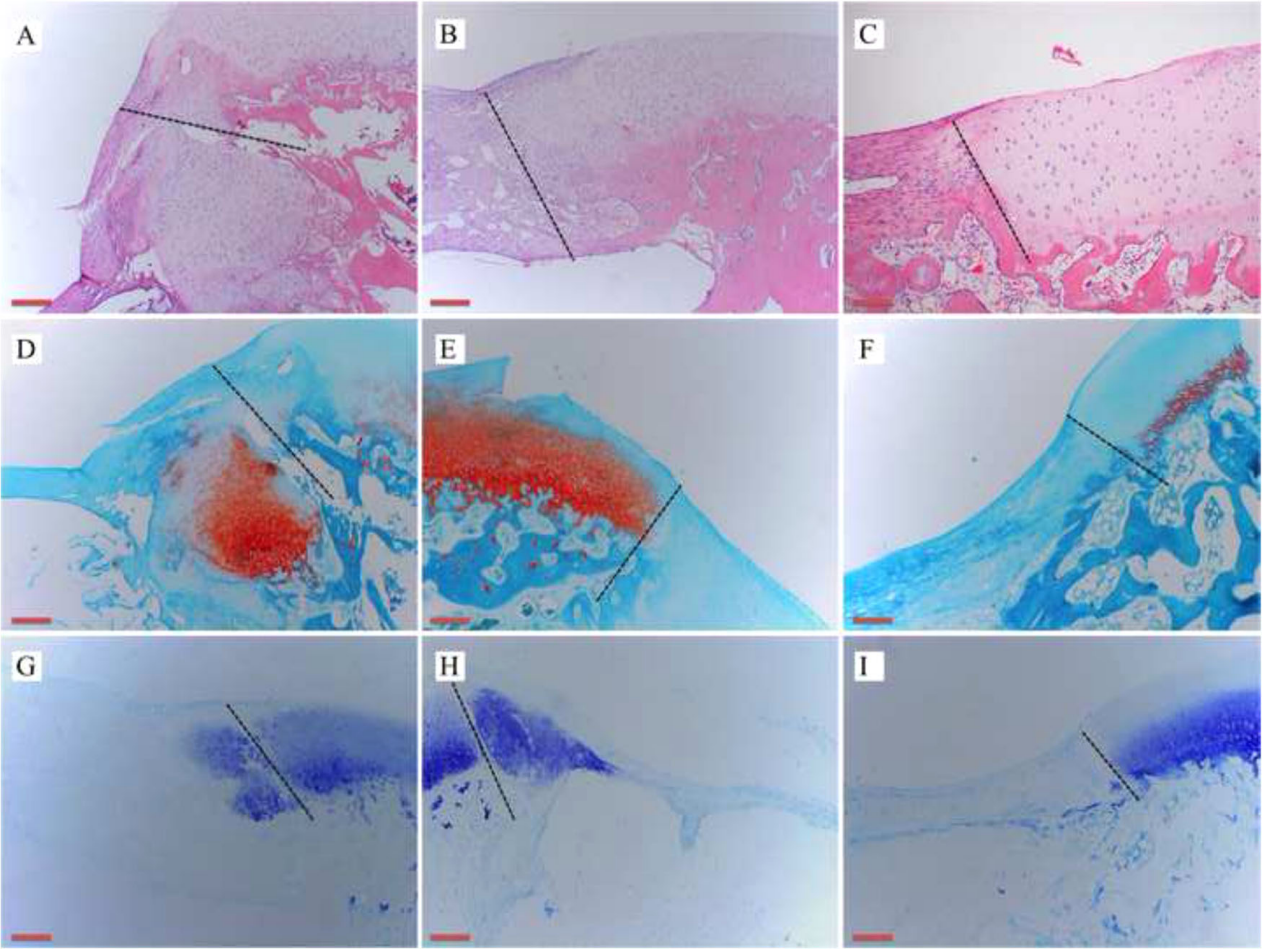
Representative histological staining after 1 month of implantation, the black dotted lines represent the boundary between implantation and natural cartilage (scale bar: 100 mm): **A–C** H&E staining results in cell-seeded group, cell-free group, and control group, respectively; **D–F** Safranin O staining results in cell-seeded group, cell-free group, and control group, respectively; **G–I** Toluidine blue staining results in cell-seeded group, cell-free group, and control group, respectively

**Fig. 9:**
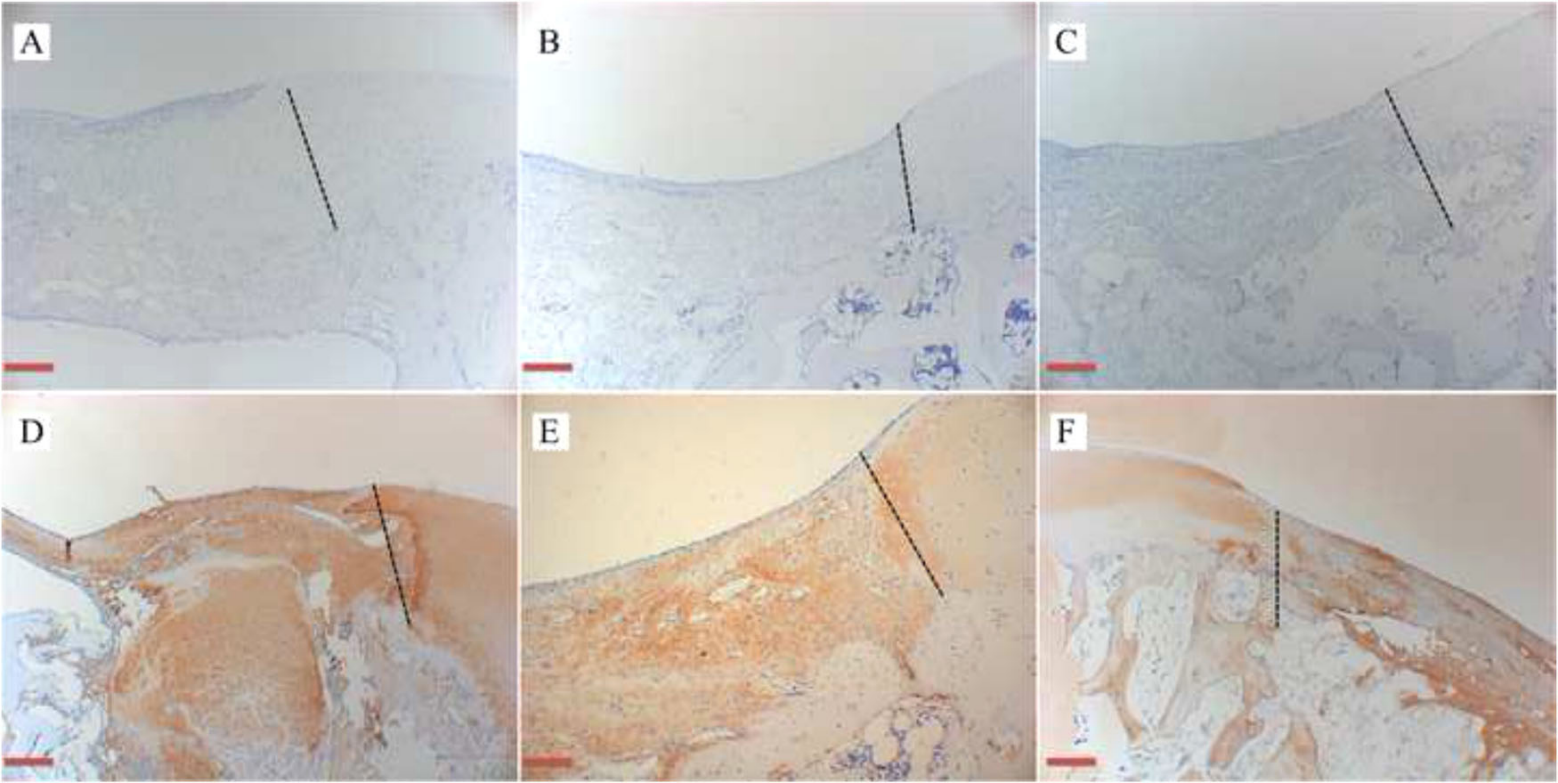
Representative IHC staining after 1 month of implantation, the black dotted lines represent the boundary between implantation and natural cartilage (scale bar: 100 mm): **A–C** IHC staining of COL II results in cell-seeded group, cell-free group, and control group, respectively; **D–F** IHC staining of COL IV results in cell-seeded group, cell-free group, and control group, respectively

General observation of the repaired goat knees showed no signs of swelling, exudation, or lumps. As shown in Fig. 7, there was no invagination or bulging of the hydrogels in the defect areas, and there was no obvious edge between the implant and surrounding normal cartilage tissue. The new cartilage-like tissue around the defect was visible and closely adhered to the surrounding tissue. H&E, Safranin O, and Toluidine blue staining showed that the chondrogenic matrix was more prominent in the cell-seeded group, followed by the Cell-free group, and only a small amount of cartilage-like matrix was produced in the control group (Fig. 8). IHC staining of COL II and COL IV was the most significant in the cell-seeded group, followed by the cell-free group, and the control group was the weakest (Fig. 9).

**Fig. 10:**
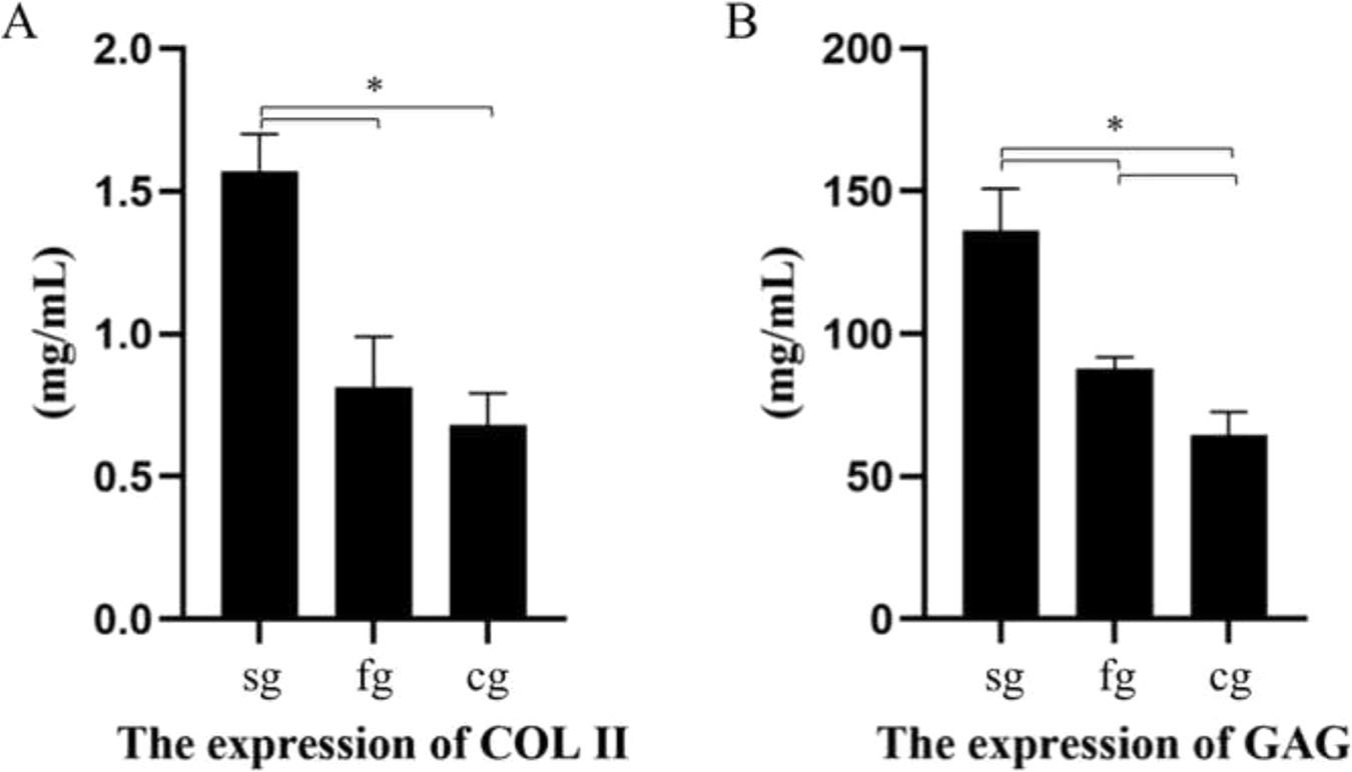
The quantitative analyses of COL II (**A**) and GAG (**B**) by spectrophotometry. (sg: cell-seeded group; fg cell-free group; cg control group; *p < 0.05)

**Fig. 11:**
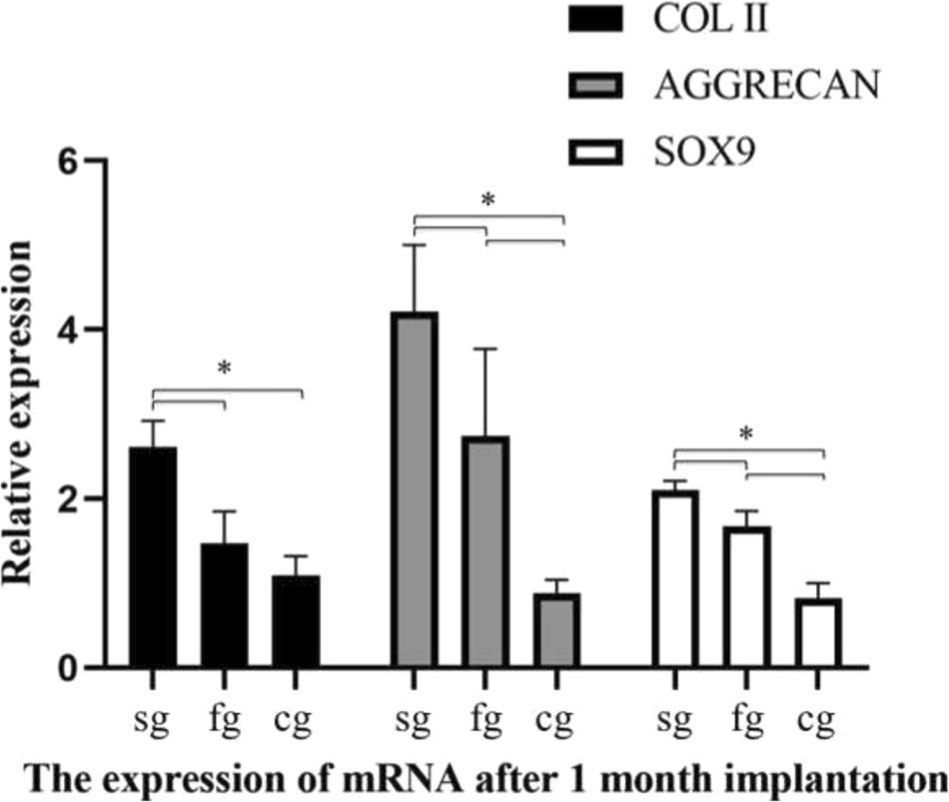
The relative expression of COL II, AGGRECAN and SOX9 by RT-qPCR. (sg: cell-seeded group; fg cell-free group; cg control group; *p < 0.05)

The quantitative analyses of COL II and GAG are shown in Fig. 10. Specifically: the expression of COL II and GAG in the cell-seeded group was significantly greater than that in the other two groups (*p* < 0.05); there was no significant difference in the expression of COL II in the cell-free group and control group (*p* > 0.05); the expression of GAGs in the cell-free group was significantly greater than that in the control group (*p* < 0.05). The mRNA expression of COL II, AGGRECAN, and SOX9 are shown in Fig. 11. Specifically, AGGRECAN mRNA in the cell-seeded group was significantly greater than that other two groups (*p* < 0.05); there was no significant difference in the expression of AGGRECAN mRNA in the cell-free group and control group (*p* > 0.05); the expression of AGGRECAN and SOX9 mRNA were the greatest in the cell-seeded group, followed by the cell-free group, and the control group was the lowest (*p* < 0.05).

## Discussion

The first objective of the present study was to demonstrate the construction of new COL-HA-PVA hydrogels. The main part of the hybrid hydrogels was formed by the HA-PVA hydrogel, and COL microscaffolds were formed in the macropores of the hydrogel, which allowed for cell adhesion and integration with peripheral native cartilage. The second objective was to determine the feasibility of the COL-HA-PVA hydrogels in cartilage repair by in vitro and in vivo experiments. Our results suggest the potential of the COL-HA-PVA hydrogels in cartilage repair.

Previous studies of PVA hydrogels suggest that more surface pore structures are formed in the freeze-thaw process rather than internal connected pores [29, 30], which suggests that 3DP can be used to construct PVA hydrogels with designed geometry and a well-defined structure [31]. In our research, the COL-HA-PVA hydrogels had an interconnected macropore structure and the macropores of the HA-PVA hydrogels contained a COL reticular structure. The interconnected macropore supports nutrient transport, while the COL micropore supports cell adhesion and subsequently cartilage ingrowth. In addition, the 3D geometry can be controlled through 3DP. The diameter of the macropore was slightly smaller than that of the inverted PVA column in our COL-HA-PVA hydrogels because of the swelling of the hydrogel [32]. Overall, the size of the macropore corresponds to the diameter of the denatured PVA column, which suggests that the macroscopic structure of the porous PVA hydrogels can be controlled by CAD [33].

Based on the in vitro experiments of the adhesion rate, RGR, and IHC, the hybrid hydrogels containing COL reticular structure significantly enhanced the viability of cell seeding, which suggests that the addition of COL improves the biocompatibility of PVA hydrogels. Various previous studies have shown that COL-based hydrogels support high cell viability [34–36]. The existence of Col, the main component of natural extracellular matrix, as a microscaffold in the hydrogel significantly increased the body surface area of cell adhesion. In addition, microstructures (such as pore size) of scaffolds can affect cell implantation and tissue regeneration [37, 38]. In our research, the COL-HA-PVA hydrogels contained more COL microscaffold, and the hydrogels were more capable of maintaining cell viability and forming cartilage in vitro when the diameter of the denatured PVA column was 1.6 mm. We speculate that the pore diameter could affect the inoculation efficiency. The COL microscaffold and seeded cells were difficult to infiltrate when the macropore of the PVA hydrogels was relatively small (1.2 mm and 1.4 mm), while the COL microscaffold pore size did not support a stable structure when the macropore was relatively large (1.8 mm and 2.0 mm). Therefore, the present study chose the hybrid hydrogel with an initial PVA diameter of 1.6 mm for experiment in vivo.

The goat was used as the animal model because the anatomical structure of the knee is similar to that of humans [39–41]. The full thickness of the knee cartilage of goats is only 2 mm, and a simple cartilage defect substitute is difficult to fix firmly [42]. In addition, cartilage damage often accompanies damage to the subchondral bone. Many studies have suggested that cartilage damage and subchondral bone should be repaired [43, 44]. A cartilage defect with a diameter up to 3 mm can be repaired without intervention [45]. Therefore, large cylindrical osteochondral defects (diameter: 8 mm, height: 8 mm) were used in our research. Based on the IHC and molecular biology in vivo analysis, the COL-HA-PVA hydrogels promoted the repair of cartilage defects, and hydrogels seeded with chondrocytes were more effective than the hydrogel only group (control). These results are consistent with other studies that have shown that a scaffold inoculated with cell has a better effect on cartilage repair [46, 47]. The microfracture of subchondral bone helps in the healing process, although most of the neo-tissue might be fibrocartilage [48, 49]. Based on the results of IHC and molecular biology, a small amount of cartilage-like matrix was produced in the control group without hydrogel, which suggests that the goat cartilage has the ability for self-repair. In addition, the chondrogenic matrix was more prominent in the cell-seeded and cell-free scaffolds in this research; compared with the cell-free group, the cell-seeded group had more cartilage formation, corresponding to published research [49, 50].

Further studies are necessary for realization of the further application of COL-HA-PVA hydrogels. First, the mechanical properties of the materials, including compression, tension, shear, and friction, need to be further tested, and the mechanical properties can be optimized by structure or composition changes [51, 52]. Second, there are some differences between the biodegradable COL reticular structure and COL in natural cartilage, and the COL microscaffold in the hybrid hydrogels tends to be structurally unstable. Further study is needed to balance the degradation of COL and the formation of neo-tissue. Finally, longer periods of observation in vivo should be performed to verify the practicability of COL-HA-PVA hydrogels in cartilage defects.

## Conclusions

COL-HA-PVA hydrogels were created using FDM printing, freeze-drying crosslinking, double steaming water demolding, and genipin crosslinking. In the hybrid PVA hydrogels, the HA-PVA hydrogels have an interconnected macropore structure, which simulates the characteristics of the viscoelastic material of natural cartilage, while the infilled degrading COL microscaffolds improved the biocompatibility of hydrogels, allowing for cell adhesion. The in vitro experiments revealed excellent cellular biocompatibility, and the hybrid hydrogels had more cartilage formation when the diameter of the print-denatured PVA scaffold was 1.6 mm. The in vivo results showed that the COL-HA-PVA hydrogels could effectively repair cartilage defects, especially when inoculated with chondrocytes in advance. The results of this study indicate that the COL-HA-PVA hydrogels have promising application in cartilage repair.
